# Optimal Spectral Wavelengths for Discriminating Orchard Species Using Multivariate Statistical Techniques

**DOI:** 10.3390/rs12010063

**Published:** 2019-12-23

**Authors:** Mozhgan Abbasi, Jochem Verrelst, Mohsen Mirzaei, Safar Marofi, Hamid Reza Riyahi Bakhtíari

**Affiliations:** 1 Faculty of Natural Resource and Earth Science, Shahrekord University, Shahrekord 8815648456, Iran; 2 Image Processing Laboratory (IPL), Parc Científic, Universitat de València, 46980 Paterna, València, Spain; 3 Environmental Pollutions, Grape Environmental Science Department, Research Institute for Grapes and Raisin (RIGR), Malayer University, Malayer 65719-95863, Iran; 4 Grape Environmental Science Department, Research Institute for Grapes and Raisin (RIGR), Malayer University & Water Science Engineering Department, Bu-Ali Sina University, Hamedan 65178, Iran

**Keywords:** field spectroscopy, orchards species, ANOVA–RFC–PCA, PLS, optimal spectral wavelengths, discriminant analysis

## Abstract

Sustainable management of orchard fields requires detailed information about the tree types, which is a main component of precision agriculture programs. To this end, hyperspectral imagery can play a major role in orchard tree species mapping. Efficient use of hyperspectral data in combination with field measurements requires the development of optimized band selection strategies to separate tree species. In this study, field spectroscopy (350 to 2500 nm) was performed through scanning 165 spectral leaf samples of dominant orchard tree species (almond, walnut, and grape) in Chaharmahal va Bakhtiyari province, Iran. Two multivariable methods were employed to identify the optimum wavelengths: the first includes three-step approach ANOVA, random forest classifier (RFC) and principal component analysis (PCA), and the second employs partial least squares (PLS). For both methods we determined whether tree species can be spectrally separated using discriminant analysis (DA) and then the optimal wavelengths were identified for this purpose. Results indicate that all species express distinct spectral behaviors at the beginning of the visible range (from 350 to 439 nm), the red edge and the near infrared wavelengths (from 701 to 1405 nm). The ANOVA test was able to reduce primary wavelengths (2151) to 792, which had a significant difference (99% confidence level), then the RFC further reduced the wavelengths to 118. By removing the overlapping wavelengths, the PCA represented five components (99.87% of variance) which extracted optimal wavelengths were: 363, 423, 721, 1064, and 1388 nm. The optimal wavelengths for the species discrimination using the best PLS-DA model (100% accuracy) were at 397, 515, 647, 1386, and 1919 nm.

## 1 Introduction

In Iran, cultivations and orchards cover about 12% of the total land area. According to the Agriculture Jihad Ministry, Iran ranks first in the Middle East and 9th in the world in fruit production. In Chaharmahal va Bakhtiyari province there are 40,890 hectares of orchards, whereby almonds, walnuts, and grapes cover most of the area with 37, 20, and 12 percent respectively. Therefore, almond (*Prunus amygdalus*), walnut (*Juglansregia*) and grape (*Vitis vinifera*) are the dominant orchard species, representing about 69% of the orchard cover in this area. Reliable and up-to-date information about composition and distribution of these orchard species is not only crucial for the economy, but is also important for managers and decision makers. Information about orchard species was traditionally obtained by means of field observation techniques. These techniques are not only labor-intensive and time-consuming, but also the obtained information may be inaccurate and incomplete due to limited accessibility [1,2]. Alternatively, remote sensing provides a time-and-cost-efficient and accurate way of obtaining data on species across wide areas [3,4]. Particularly optical remote sensing devices reached maturity, with a diversity of sensors covering a wide range of spatial and spectral resolutions. Both types of multispectral and hyperspectral remote sensing data have been used for discrimination and classification of vegetation [5–7]. However, many researchers indicate that multispectral data such as Landsat and SPOT images produce general land cover classifications that are too broad to be utilized for identification of orchards species. Discrimination of subtle differences in species composition remains a major problem of these systems [8–10]. Consequently, the application of airborne imaging spectroscopy information in conjunction with field spectroscopy is essential in orchard discrimination at the species level [11–13]. A spectroradiometer records electromagnetic energy reflected from leaves in hundreds of narrow, contiguous spectral wavelengths, leading to hyperspectral data, which may allow the discrimination of different species [5,14–16]. Moreover, hyperspectral data can be used to discriminate species varieties [17,18], or to assess the health status of vegetation [19,20], water amount in the plant bodies [21,22], biomass status [23], quantity and quality of crops [15,24,25], plant pests and diseases [26,27] and contaminations [28,29]. In fact, a field spectroradiometer is able to record a unique spectral curve (spectral fingerprint) for any object [18]. These spectral signatures can then be brought together into a spectral library, and so contribute to the remote sensing community with web-based platforms and enhanced data browsing/search capabilities [18,30–32]. Accordingly, by using field spectroscopy a large amount of data is obtained in the form of spectral curves, that in turn can be analyzed to identify the desirable objectives [16,33,34].

One issue in the analysis of hyperspectral data is the processing of large quantity data as obtained from numerous wavelengths [17,29]. The so-called multicollinearity problem is commonly found in spectral data because of high correlations usually occurring along many wavelengths, particularly adjacent ones [35]. Therefore, several methods of statistical analysis such as partial least square (PLS) are commonly employed to eliminate redundant variables in the original data [36–41]. PLS is a standard calibration method for analyzing spectral data and figuring out the optimum number of necessary wavelength for detecting the spectral differences of vegetation in hyperspectral or spectroscopy studies [18,29,42,43]. Preisner et al. [44] compared partial least squares discriminant analysis (PLS-DA) to other methods such as principal component analysis (PCA). They concluded that PLS-DA was more adequate than the other two methods at the species level.

Selection of optimal spectral region can mitigate the curse of dimensionality and improve the classification precision significantly [34,45]. For instance, Mureriwa et al. [46] identified invasive plant species using field spectroscopy techniques in the north Virginia using field spectroscopy and guided regularized random forest in order to separate Prosopis glandulosa from co-existent species. Adam and Mutanga [34] used analysis of variance (ANOVA) and classification regression tree in determining optimal wavelengths for the differentiation of Papyrus from other species in South Africa. Finally, Baldeck et al. [16] compared two leading single-class classification methods—binary support vector machine (SVM) and biased SVM—for their performance in identifying pixels of a single focal species. Additionally, for the quantification of leaf traits, Feilhauer et al. [47] tested if an ensemble of regression models, consisting of PLSR, random forest, and SVM regression models, is able to improve the robustness of the spectral band selection process as compared to the outcome of a single technique.

Over the past years, spectral characteristics of species have been extensively studied and used as reference information for imaging spectroscopy applications [48–50]. These spectral data can be further used in modeling approaches to determine the fraction of photosynthetic vs. non-photosynthetic materials [51], to detect large scale pigment shifts in plaint functional groups, to identify invasive species, serving as ground truth for remote dominant sp ec i es determination, as wel l as upscaling efforts using radiative transfer modeling [52]. Monitoring and mapping of various orchard species using hyperspectral images requires a proper understanding of specific spectral behavior of each species.

Altogefher, the objectives of this siudy were first to acquire spectral fingerprints of dominant orchard species in Chaharmahal va Bakhtiyari province of Iran using field spectroscopy at leaf ievel, inchiding almond (*Prunus amygdalus*), walnut (*Juglans regia*), and grape (*Vitis vinifera*). The recond objective was identifying the wavelengths (in the range of 350 to 2h00 nm) with highest sensitivity and performance [o separate these species. To reach these two objectives, two methods were employed using the spectral fingerprints prepared for main orchard species, one includes three steps; ANOVA, random forest classifier (RFC) and PCA, and the second is the standard PLS method. For both methods, we subsequently established whether the orchard species were spectrally distinguishable using discriminant analysis (DA) in osder to finally consolidate the optimal wavelengths and discriminate the plant species.

## 2 Materials and Methods

### 2.1 Study Area

**Figure 1:**
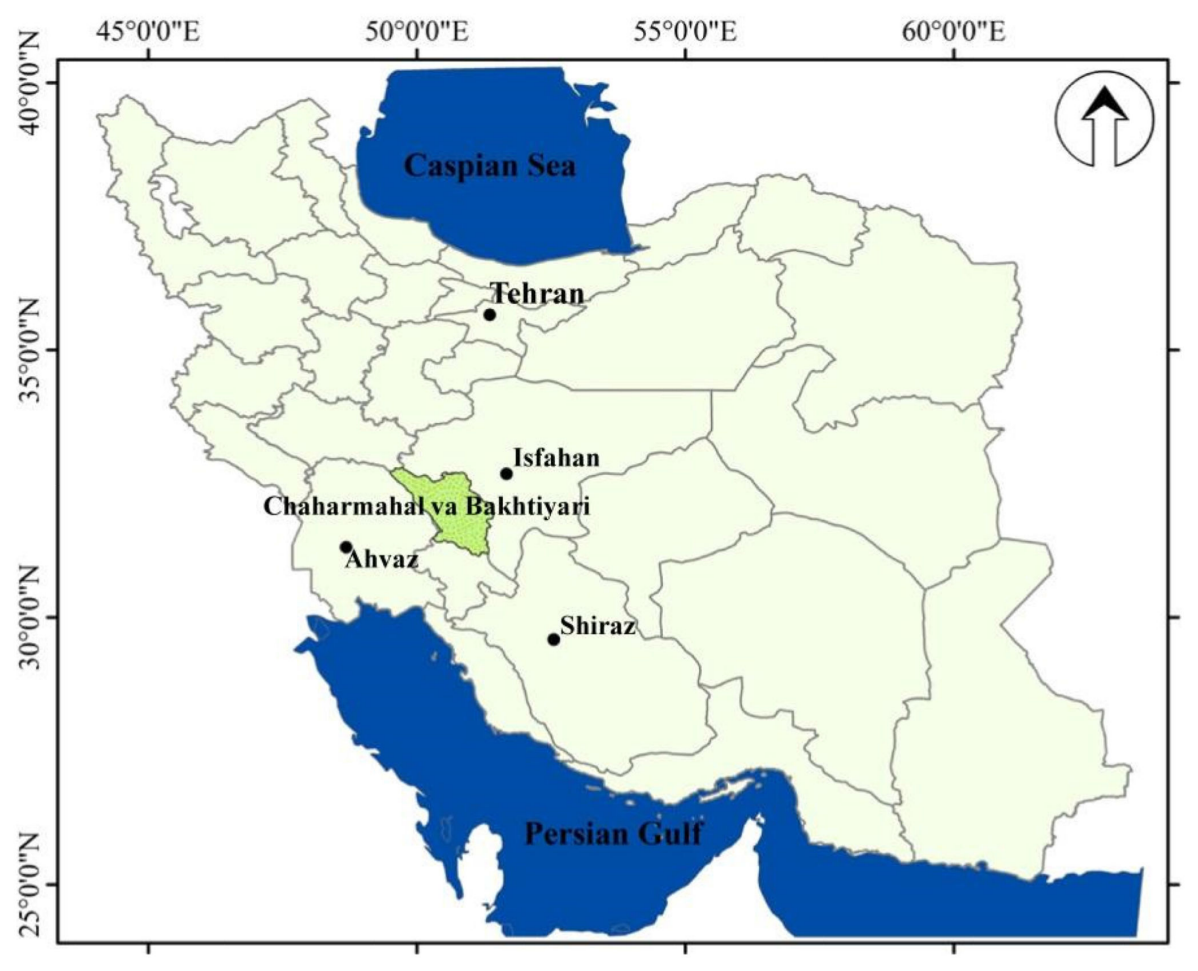
Location of study site, Chaharmahal va Bakhtiyari, Iran.

Chaharmahal va Bakhtiyari is one of the coldest provinces in the western of Iran, which it located in 39°10’00” to 32°50’00”N latitude and 49°30’00” to 52°25’00’? longitude (Figure 1). Monthly mean temperatures range from 3 °C in February to 30 °C in July. Mean annual rainfall corresponds to 600 mm per year [53,54].

### 2.2 Field Spectral Acquisition

Vegetation classification at the species level benefits from introducing phenological and biochemical information to spectral libraries [49]. For classifying at the species level it is therefore necessary to build spectral libraries across a wide range of situations. We designed s field measurement campaign from different leaf samples of the species varieties lhat support specific structural (morphological and biochemical) characteristics [55]. Thus, to create a diverse spectral library of orchards planls, multiple leaf samples were collected to fully capture variation in plant communities [21]. The number of treatments for grapes, almonds and walnuts were 10, 10, and 13 respectively. In each treatment five tree stands were sampled, meaning that in total of 165 spectroscopy samples were performed, and spectral curves were prepared for further analysis.

In order to obtain spectral curves in the range of 350 to 2500 nm, the ASD FieldSpec® 3 spectroradiometer was used. The device has a spectral resolution of 3 and 10 nm and a sampling interval of 1.4 and 2 nm for the spectral regions of 350–1000 nm and 1000–2500 nm respectively, which are automatically interpolated to 1 nm intervals by this instrument [56]. The wavelength configuration of the spectroradiometer are organized as the visible (VIS: 350–700 nm), the near infrared (NIR: 700–1350 nm) and the shortwave infrared (SWIR1: 1350–1800 nm and SWIR2: 1800–2500 nm) wavelengths [57]. The probe was held at a distance of 60 cm above the pile of leaves (25° FOV; diameter 26.59 cm). The spectral measurements were carried out on a large matt black in a completely dark room laboratory with a special bulb light to eliminate the effect of water vapor, temperature, wind, and other environmental interferences errors. Finally, in order to minimize instrument noise, 100 scans were averaged for every single spectral measurement. Measurement noise introduced by variation in the atmosphere between the reference panel and target measurement were minimized by keeping time between samples as short as possible. Recalibration was performed at least every 15 min. The obtained curves were initially reviewed. In cases where the obtained curves were inconsistent with the normal plant curves they were discarded and the spectral measurement was repeated.

### 2.3 Identifying Optimal Wavelengths

The collected spectral data consisted of 2151 wavelengths ranging from 350 to 2500 nm. Selection of optimal spectral regions for separating studied plant species (grape, walnut and almond) was obtained by using multivariate statistics techniques. Two methods were exploited in this study to identify optimal wavelengths: one; including three-stage approach called ANOVA–RFC–PCA, and the second, PLS.

#### 2.3.1 ANOVA–RFC–PCA Method

In the first step, ANOVA was used at 95% and 99% confidence level (CL) (i.e., *p <* 0.05 and *p <* 0.01, respectively) with a post-hoc Scheffé test [34], to identify wavelengths with different average spectral values in all studied species. Due to the large number of selected wavelengths at the first stage the results of 99% confidence level were used and reported. In the second step RFC was used to evaluate the strength of the wavelengths in the classification of almond, walnut and grape species. Here RFC inputs include the selected wavelengths in the previous stage (ANOVA) as independent variables, and plant species (walnuts, almonds, and grapes) were considered as the response variable. Therefore, at this stage, wavelengths were identified that had significant differences in the obtained curve of almonds, walnuts, and grapes. In this step, the optimal wavelengths in species separation were selected based on variable importance (VI) statistics [58]. The RF is a decision tree ensemble method based on bagging and random subspace [59–61], which can be used for analyzing high-dimensional data. RFC uses multiple trees to train and predict a sample. This model randomly resamples *k* samples with replacement from the original training sample set to generate a new training sample set using a bootstrap resampling technique.

At the end of first and two steps, a set of wavelengths were identified that have both different mean values and high ability to classify the studied species. Because these selected wavelengths may be correlated, then in the third step, the main components analysis (PCA) was used to introduce the optimal and final wavelengths. PCA is one of the multivariate statistical techniques, and when dealing with a large amount of data, it can function as an appropriate method for dimensionality reduction [62]. The suitability of the data for PCA was evaluated by the Kaiser–Meyer–Olkin (KMO) test. A high KMO value (close to 1) generally indicates that PCA results may be useful [63], which is the case in this study: KMO = 0.93. In order to achieve a better separation inputs in the components, the Varimax rotation method was used [64,65]. PCA reduces the dimensions of the spectral dataset by explaining a large part of the variance using synthetic factors, called principal components (PCs). Therefore, the whole range of wavelengths can be compressed into the first few PCs, which explain the largest amount of the variance of the spectral dataset [66]. Finally, the main variables in each component are determined based on the maximum factor load [67].

#### 2.3.2 PLS

Alternatively, PLS was employed as a classifier method to reduce the dimension of hyperspectral data and select the optimal wavelengths for classifying the studied species. This method is based on linear least squares regression that performs new components instead of the original input data. The X variables (the predictors) are reduced to principal components, as are the Y variables (the dependents). The components of X are used to predict the scores on the Y components, and the predicted Y component scores are used to predict the actual values of the Y variables [18]. The main advantage of PLS compared to PCA is that, first the response variables are also considered in PLS in parallel with the dimensionality reduction and the overlapping elimination [40,42]. Second, while the unsupervised nature of the PCA algorithm provides a means to achieve unbiased dimensionality reduction, PLS discriminant analysis that relies on the class membership of each observation will be applied as a supervised form of discriminating analysis [68]. Other capabilities of PLS are the ability to analyze highly collinear and high-volume spectroscopy data, providing a regression model between independent and dependent variables and also the acceptable speed of processing [37,69]. After PLS is implemented some components are formed, which each of them explained a part of variance. The degree of correlation between independent variables and components is represented by factor load. Therefore, the factor load of wavelengths in each component was used for selecting optimal wavelengths to discriminate orchard species [40,41]. Any wavelength that had maximum factor load in the each developed principal component was chosen as the representative of that component [29]. The model was run whereby the wavelengths were considered as independent variables and the class membership information of three orchard species were assumed as dependent variables, which coded in matrix form into Y.

#### 2.3.3 Accuracy Assessment

Discriminant analysis (DA) is a parametric statistical method that is applied to classify input data into two or more groups. The usage of DA in species separation studies is common because the response variable in this method is categorical [17,70]. DA can also be used as a discriminant analysis to investigate how variables contribute to group separation and to place objects or individuals into defined groups. Cross-validation was applied in the DA in order to check the performance of the discrimination. This technique is used to compensate for an optimistic apparent error rate [17]. In the training step, DA was carried out with 70% of the data and the structure of the model was saved for testing step, then in the testing step, performance evaluation was performed with the remaining data [18].

## 3 Results

### 3.1 First Method: ANOVA–RFC–PCA

**Figure 2:**
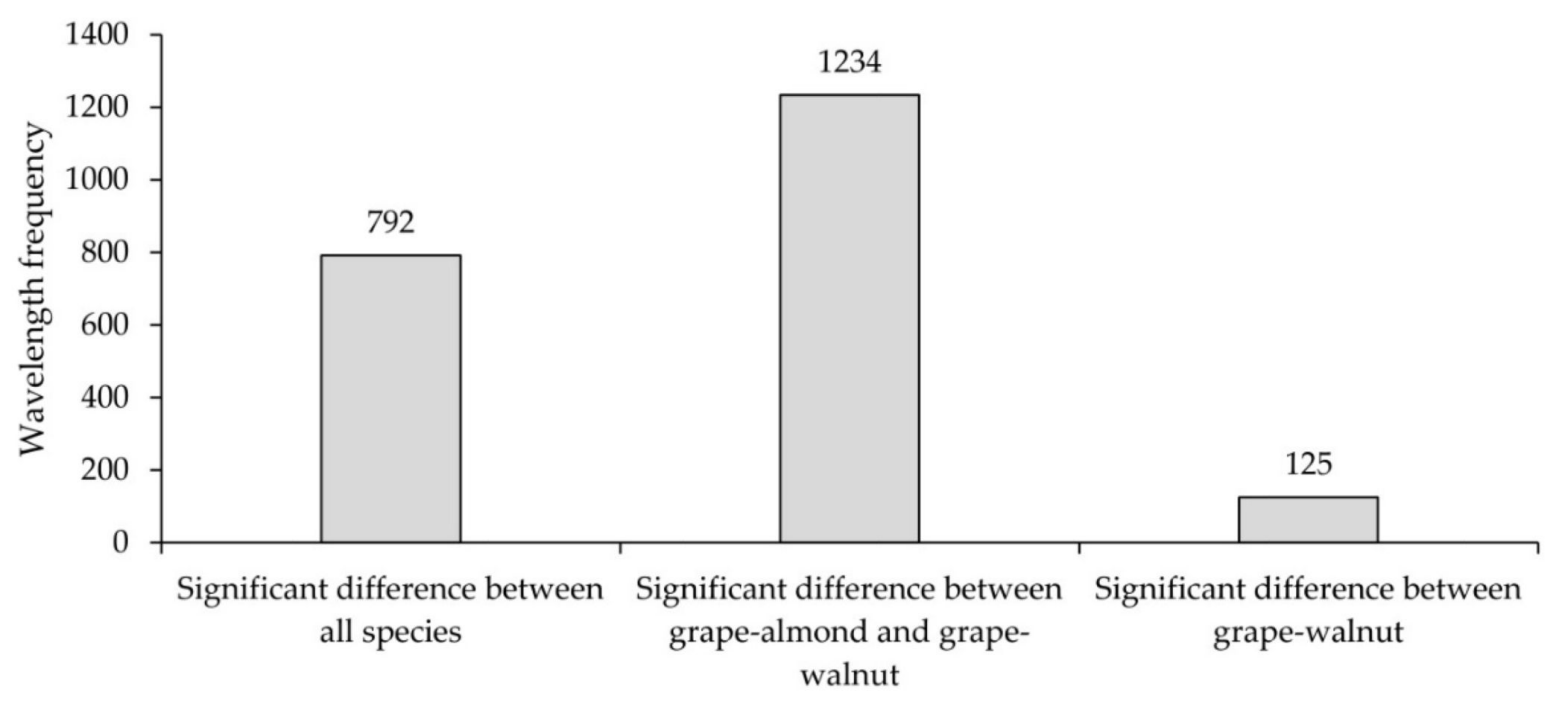
Frequency of wavelengths that have the potential for separating walnut, almond, and grape species at the leaf surface in accordance with the ANOVA test (Vertical apis: wavelengths frequency and horizontal axis: wavelengths and status).

ANOVA was applied to identify the optimum wavelengths with distinct spectral behavior in the studied species. 2151 wavelengths (from 350 to 2500 nm) were analyzed and the result was reported at confidence level of 99%. The ANOVA results indicate that 792 wavelengths were able to separate the grapes, almonds, and walnuts species from each other (Figure 2). The residue of the wavelengths (2151-792 = 1359) were analyzed for pairwise separation of species. The results indicate that 1234 wavelengths showed a significant difference between grapes–almonds and grapes–walnuts (Figure 2). Additionally, ANOVA results indicate that 125 wavelengths were only able to separate grapes and walnuts species (Figure 2). In the SWIR region, most wavelengths are able to distinguish between grape-almond and grape–walnut species.

**Figure 3:**
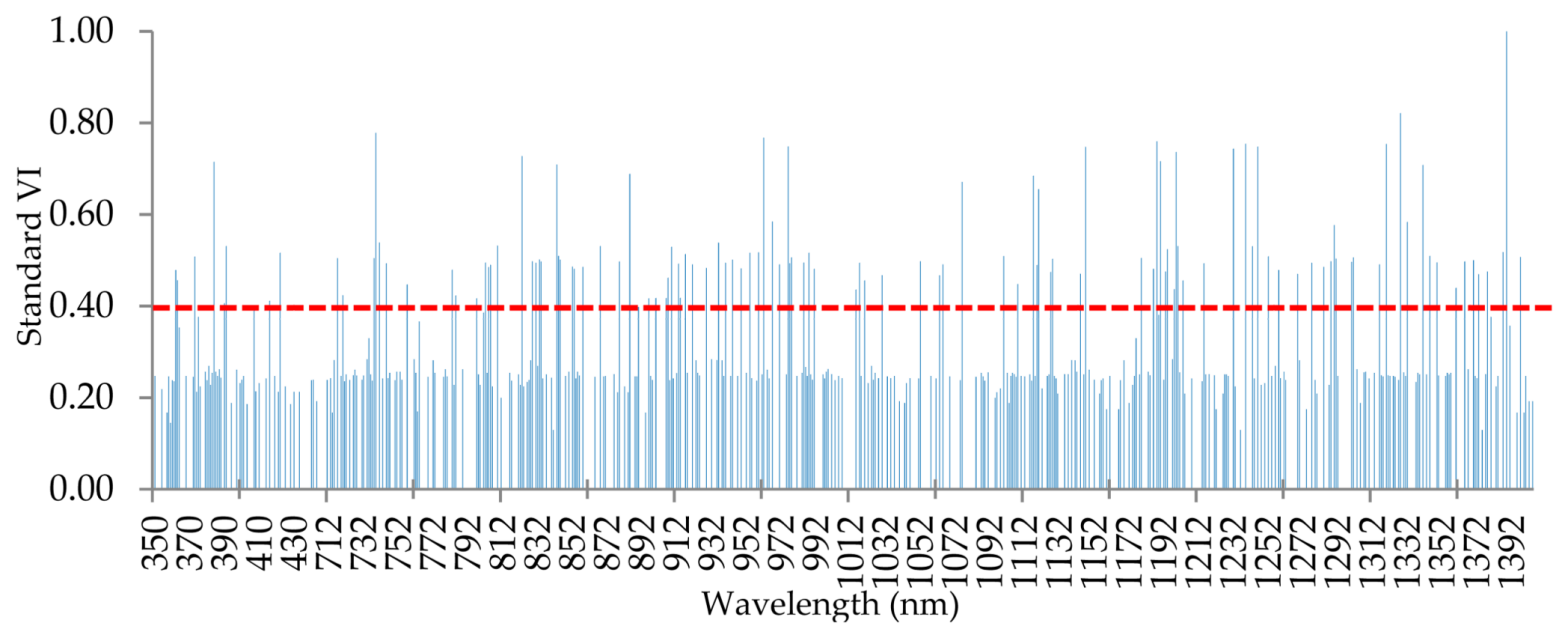
The average of spectral curves and the spectral distribution of the wavelengths in terms of strength in the separation of studied species at the leaf level (at 99% confidence level); Class *a*: Significant difference between all species, Class *b:* Significant difference between grape–almond and grape–walnut; Class *c:* Significant difference between grape and walnut.

In Figure 3, the mean spectral gradients of walnut, almond and grape samples are shown at the leaf level, which are taken from all treatments and rep licates (the average of 65, 50, and 50 samples respectively). In this figure, the distributions of spectral wavelengths in the range of 350 to 2500 nm are displayed for separation of the studied species. The results presented in Figure 3 are based on an ANOVA test at a confidence level of 99%.

In accordance with Figure 3, in class ***a***, wavelengths emerge where all the studied species (almond, walnut, and grape) express a distinct spectral behavior, and so are considered as the selected wavelengths for the second step (identification of effective wavelengths in species classifying). This class is located at the beginning of the visible region (from 350 to 439 nm), as well as the NIR region (from 701 to 1405 nm). While, in class ***b*** (from 440 to 700 nm), for some wavelengths grape–walnut and grape–almond express distinct spectral behavior but the spectral behavior of almond–walnut is similar in this spectral region (Figure 3). In most wavelengths in the SWIR region-class ***b*** (1405 to 1890 and 1978 to 2455 nm), grape–almond and grape–walnut species lead to a significant different mean (at a confidence level of 99°%). Walnut–almond species in class ***b*** behave spectrally alike (Figure 3). Additionally, in parts of tha SWIR class *c* (1891 to 1977,2456 to 2457, and 2463 to 2500 nm) wavelengths emerge that can only separate the grape and walnut species from each other, while the spectral response of almond and walnut species is similar for these regions (Figure 2).

**Figure 4:**
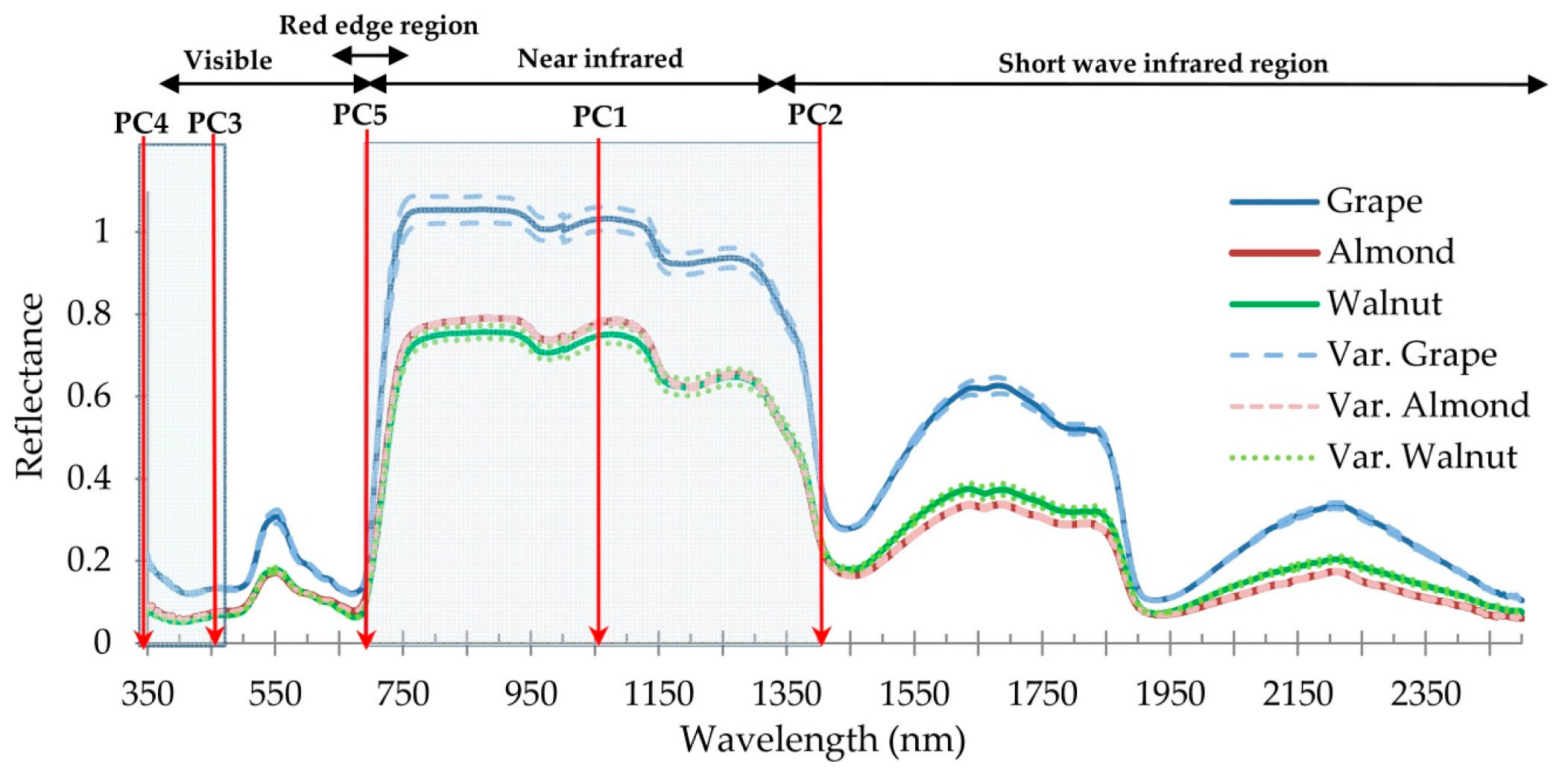
The results of random forest classifier (RFC) in introducing the most important wavelengths (*n* = 118 where VI > 0.4), for the classification of almond, walnut, and grape species based on VI.

According to Figure 2, it can be admitted that in the first step all studied species (walnut, almond, and grape) expose a distinct spectral behavior in the 792 wavelengths (at a confidence level of 99%). Since the number of these wavelengths is very high (792 wavelengths), it is necessary to identify the most suitable wavelengths for species classification. Therefore, these 792 wavelengths were considered as RFC inputs. Some of the results obtained from RFC, i.e., the number of selected wavelengths and their importance for the separation of walnut, almond and grape species are shown in Figure 4. According to this result, 118 spectral wavelengths appear to be most promising (VI > 0.4) in the classification of studied species.

After conducting the first and second steps, spectral wavelengths were identified where the species (almond, walnut and grape) cause a distinct spectral behavior, which are important for accurate classification. In the third step, we aimed to reduce the overlap between the wavelengths and reduce their numbers by using PCA. According to the PCA results, the first five components were able to explain 99.87’% of variance. The factor loah of the most important wavelengths in the first to fifth components is shown in Figure 4. According to this result, fire wavelengths 1053,1064, and 1077 nm led to the highest load factor in the first component from the 118 wavelengths entered into the PCA.

**Figure 5:**
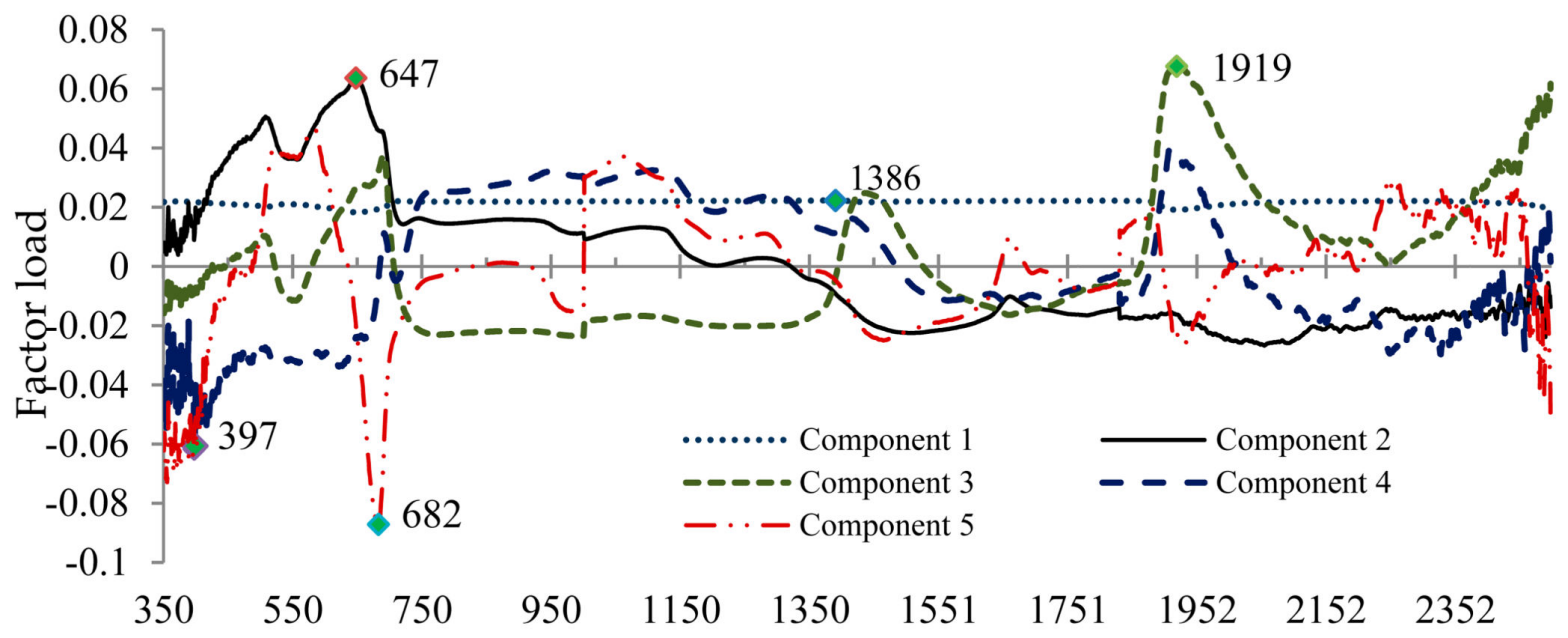
The position of five optimal/final wavelengths in the separation of walnut, almond, and grape species in the studied area, introduced by PCA (vertical axis: spectral reflectance, and horizontal axis: spectral wavelengths).

In the second component, the wavelengths 1379, 1388, and 1390 nm, in the third component, wavelengths 423, 423, and 422 nm, in the fourth component, wavelengths 363, 364, and 37 4 nm and in the fifth component, 721, 718, and 739 nm led to ‘he most factor load in this study (Table 1). Therefore, in general, the waveleng ths o f 363, 423, 721, 1064, and 1388 nm can be cons i dered as optimal wavelengths for discriminating studied orchard species. Additionally, in order to clarify the PCA results, in Figure 5 the optimal five-wavelength positioning introduœd by this analysis was shown for the separation of walnut, almond and grape species in the range of 350 to 25b0 nm. According to Figure 5, the first component is dominant in the NIR, the second component is dominant in the SWIR, the third and fourth components are within doe visible region, and the üfth component is in the red edge region.

### 3.2 Second Method: PLS

**Figure 6:**
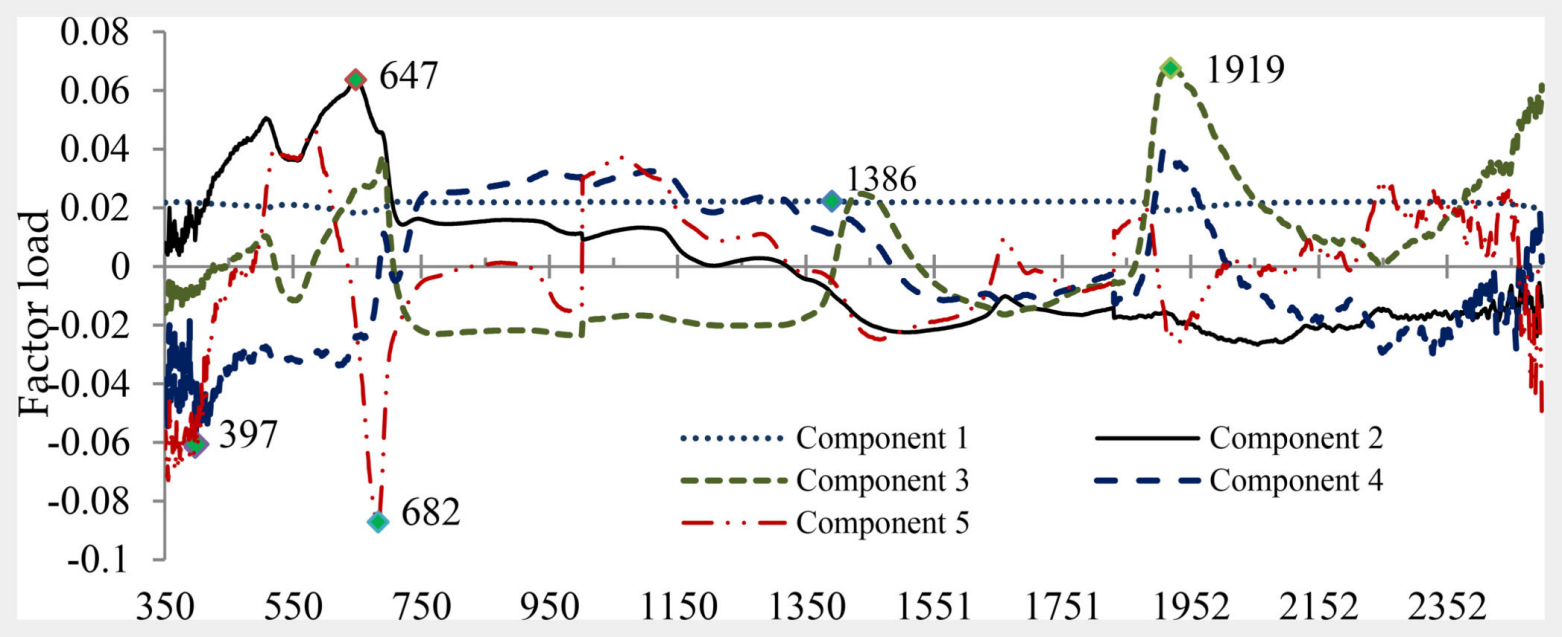
The loading of wavelengths in the first five extracted components by PLS.

As second experiment, all the wavelengths (350–2500 nm) were entered into PLS at leaf spectral reflectance as X variable and three species as dependent variables. The loading of wavelengths in the first five extracted components by PLS are shown in Figure 6. The results of the best model show that the six most important wavelengths were the optimal spectrum in discriminating the species (Table 2). They all fell in the range 390–690 nm, the visible (blue, green to red) and two wavelengths at 1386 and 1919 nm the infrared range, suggesting that photosynthetic pigments, water, and biochemical content are the most important variables determining spectral separability of the studied species. The PLS accuracy results show that all three species were highly spectrally distinguishable, mainly in the VIS region of tire spectrum.

### 3.3 Accuracy Assessment

Considering the difference in model accuracies between the ANOVA–RFC–PCA and PLS (Table 2), better overall accuracy (OAA) was obtained for PLS (OAA = 100%) than ANOVA–RFC–PCA (OAA = 95.6%). Although both the ANOVA–RFC–PCA and the PLS-DA model achieved good results for discriminating the studied species, but the PLS-DA model yielded a slightly superior OAA than ANOVA–RFC–PCA.

## 4 Discussion

In the present study, leaves of all orchard species reflect a typical spectral curve without significant influences of environment interferences e.g., water vapor. This indicates a good quality of the device (ASD FieldSpect) used for the spectral acquisition and stable conditions in the laboratory environment [69]. A field spectroradiometer has been used in several studies to determine the optimal wavelengths for separation plant species at in situ and in vitro measurements [34,45,66,71–74]. One of the most important issues in this field is data reduction and introducing optimal wavelengths. In this regard, a small number of wavelengths must be selected to provide in-depth information, while at the same time missing data must be minimized [5]. The results of this study support the feasibility of using a field spectroradiometer as a nondestructive technique for detecting orchard species without considering tedious biochemical measurements.

Two methods were conducted to find optimal wavelengths for the separation of walnut, almond, and grape species in Chaharmahal va Bakhtiyari province. In the first used method, three stages were applied; an ANOVA test was first used to determine all wavelengths where the spectral behavior of species varied significantly and had the potential to be selected as the optimal wavelengths. The results of ANOVA at 99% confidence level suggested that 792 wavelengths have the primary potential for separating of walnut, almond, and grape species from each other. This confidence level indicates a major significant difference in spectral behavior of the studied species with the least error.

In the ANOVA result, all of the 2151 wavelengths in the 350–2500 nm range showed significant differences for at least two species. Closer spectral similarity appeared befween the almond and walnut species, while the grape spectral behavior was always different. The s difference could be due to the intracellular and extracellular structure of the leaves, the concentration of biochemical substances including chlorophyll, carotenoid, nitrogen, and water in the plant species, as has also been mentioned in related studies [4,17,34,45].

The foliage of all studied orchard species exposed a distinct spectral behavior at the beginning of the visible region (from 350 to 439 nm), as well as in the red edge and NIR (from 701 to 1405 nm). In comparison with other studies, Schmidt and Skidmore [14] observed different spectral behavior in the visible region, Adam and Mutanga [34] and Vaiphasa et al. [71] similarly achieved spectral differences in the red edge region. Aneece and Epstein [45] and Thenkabail et al. [75] also found the NIR wavelengths as optimal wavelengths for plant species differentiation. It is worth noting that in this study the highest frequency of optimal wavelengths was observed in the red edge and NIR region (wavelengths number: 721, 1064, and 1388 nm). As with these findings, several related studies that investigated the spectral differences between plants species found similar key wavelengths located in the red edge and NIR region [34,71,75].

Although foliar biochemical parameters were not measured in this study, it can be deduced that the difference in spectral behavior in the red edge could be due to differences in the levels of chlorophyll, carotenoid, nitrogen, and water [76]. On the other hand, the significant difference between the spectral wavelengths in the NIR region is caused by the differences in the leaf structure of the plant species [14,57].

Here, 2151 wavelengths were analyzed for the discrimination of dominate orchard species (walnuts, almonds, and grapes) in Chaharmahal va Bakhtiyari province, and after the ANOVA test, 792 wavelengths with significant differences (at a confidence level of 99%) in all three species were introduced to RFC. The RFC possesses attractive capabilities for spectroscopy data processing such as high classification accuracy, capability for analyzing huge volume of data, managing multiple variables and providing an estimation of the most important variables in the classification [60]. RFC was here used to identify wavelengths that played the most role and efficiency in plant species discrimination. This approach enabled to reduce 792 input wavelengths to 118 important wavelengths in classifying the target species. This finding demonstrates the validity of the RFC method, and it also can be recommended as an appropriate approach for hyperspectral remote sensing studies [77–79]. Since 118 wavelengths provided similar information in many cases and they correlated with each other, it was necessary to use PCA to reduce the complexity of the data. The PCA compressed the spectral variability in five components, which represented 99.87% of the variance. Therefore, five wavelengths were introduced as optimal wavelengths that caused the maximum factor load in each component. The identified optimal wavelengths, i.e., 363, 423, 721, 1064, and 1388 nm, were compared in Table 3 with selected wavelengths in other similar studies. The encountered difference in the location of optimal wavelengths as a function of plant species can be attributed to the differences in the amounts of pigments, optical components, and biochemical properties in the plants leaf structure, which leads to distinct spectral reactions in the same spectral range [14,57,80].

## 5 Conclusions and Recommendations

Based on the spectral analysis of foliage from dominant orchard tree species (almond, walnut and grape) in Chaharmahal va Bakhtiyari province, it can be concluded that hyperspectral field spectroscopy at leaf level can accurately spectrally discriminate these species. Moreover, field spectroscopy is easy to use, rapid, eco-friendly, nondestructive, and less expensive as opposed to other approaches such as morphological-physiological technique [81], isoenzymes chemistry, and DNA analysis [82]. Specific conclusions are:

1. The combination of ANOVA, RFC, and PCA can reduce the complex dimension of hyperspectral remote sensing data.
2. The near infrared and red edge regions have played important roles in the introduction of optimal wavelengths for discriminating of plant species, which indicates the sensitivity and applicability of these spectral regions for discrimination targets.
3. Similarity has been observed in the spectral behavior of walnut and almond species, and fewer wavelengths were able to discriminate these two species. While spectral behavior of grapes leaves was more distinctly separated from walnuts and almonds, and more wavelengths had the potential for separating grapes from almonds and walnuts.
4. The PLS method showed superior and easier potential for discriminating the species as opposed to ANOVA–RFC–PCA approach.

The key wavelengths extracted in this study indicate important sensitivities towards plant characteristics such as pigment types, moisture, and cellular structure of the plant, and eventually can be used to estimate these variables from hyperspectral imagery. Results of this and related studies are the prerequisites for aerial and satellite remote sensing surveys, and reflects the high performance of hyperspectral imagery for species discrimination targets. We therefore recommend to carry out similar studies to determine optimal wavelengths for multiple orchard species. With the availability of imagery data, it is possible to obtain relevant and accurate information from the orchard fields in a short time. Yet, there are some challenges related to the exploitation of hyperspectral imagery for species discrimination. The first point is that image acquisition and field spectroscopy should be performed simultaneously, which can be challenging to coordinate in case of satellite hyperspectral imagery.

The second point is the problem of mixed pixels in images. For trees with open crowns, like almonds, recorded pixel reflectance is the result of interactions with leaves and branches. Additionally, for grapes, which are planted in the rows, pixels are composed of mixtures of vegetation and background soil. A solution to alleviate these mixed pixels problems could be to fly lower by using airborne hyperspectral or multispectral UAV acquisitions. Particularly the latter proved to be beneficial for orchard species mapping [83].

## Figures and Tables

**Table 1:** Principal component analysis (PCA) results; factor loads of the most important wavelengths in the first five components with Varimax rotation method.

Wavelength	Principal Components				
	PC1	PC2	PC3	PC4	PC5
363	0.51	0.52	0.67	0.13	–0.01
364	0.49	0.55	0.66	7.12	–0.02
374	0.54	0.52	0.65	0.11	–0.01
422	0.54	0.42	0.72	–0.08	0.01
423	0.53	0.43	0.73	–0.08	–0.01
721	0.60	0.49	0.59	–0.05	0.2
718	0.61	0.5	0.58	–0.01	0.17
739	0.61	0.51	0.58	–0.01	0.16
1053	0.72	0.52	0.47	–0.05	0
1064	0.72	0.52	*0.47*	–0.05	0
1077	0.72	0.51	*0.47*	–0.05	0
1379	0.49	0.72	0.46	–0.03	0.08
1388	0.50	0.72	0.46	–0.02	0.08
1390	0.50	0.72	0.48	–0.02	0.07

**Table 2:** A summary of the results obtained from the LDA in comparing of partial least squares (PLS) and ANOVA–RFC–PCA methods.

Feature Selection Method	Selected Inputs	Train	Test
		OAA %	OAA %
ANOVA–RFC–PCA	B363, B423, B721, B1064, B1388	100	95.6
PLS	B397, B515, B647, B682, B1386, B1919	100	100

**Table 3:** The frequency of selected spectral wavelengths for the separation of plant species in the four-dimensional range defined by Kumar et al. [57].

Spectral Regions (nm)	Reference	Optimal Selected Wavelengths (nm)
Visible region (350–700)	This study	363, 423
	Schmidt and Skidmore [14]	404, 428
	Adam and Mutanga [34]	No wavelength
	Aneece and Epstein [45]	350 to 399 and 500 to 549
Red edge region (680–750)	This study	721
	Schmidt and Skidmore [14]	No wavelength
	Vaiphasa et al. [71]	720
	Adam and Mutanga [34]	745, 746
	Lehmann et al. [66]	675 to 710
	Aneece and Epstein [45]	700 to 749
Near infrared region (700–1300)	This study	1064, 1388
	Schmidt and Skidmore [14]	771
	Adam and Mutanga [34]	892, 932, 934, 958, 961, and 989
	Aneece and Epstein [45]	900 to 949
Short wave infrared (1300–2500)	This study	No wavelength
	Schmidt and Skidmore [14]	1398, 1803, and 2183
	Adam and Mutanga [34]	No wavelength
	Lehmann et al. [66]	1360 to 1450 and 1630 to 1740
